# The User Knows What to Call It: Incorporating Patient Voice Through User-Contributed Tags on a Participatory Platform About Health Management

**DOI:** 10.2196/jmir.7673

**Published:** 2017-09-07

**Authors:** Annie T Chen, Rachel M Carriere, Samantha Jan Kaplan

**Affiliations:** ^1^ Department of Biomedical Informatics and Medical Education University of Washington School of Medicine Seattle, WA United States; ^2^ EBSCO Information Services Ipswich, MA United States; ^3^ School of Information and Library Science University of North Carolina Chapel Hill Chapel Hill, NC United States

**Keywords:** collaborative tagging, folksonomy, knowledge organization, self-management, body listening, body awareness

## Abstract

**Background:**

Body listening, described as the act of paying attention to the body’s signals and cues, can be an important component of long-term health management.

**Objective:**

The aim of this study was to introduce and evaluate the Body Listening Project, an innovative effort to engage the public in the creation of a public resource—to leverage collective wisdom in the health domain. This project involved a website where people could contribute their experiences of and dialogue with others concerning body listening and self-management. This article presents an analysis of the tags contributed, with a focus on the value of these tags for knowledge organization and incorporation into consumer-friendly health information retrieval systems.

**Methods:**

First, we performed content analysis of the tags contributed, identifying a set of categories and refining the relational structure of the categories to develop a preliminary classification scheme, the Body Listening and Self-Management Taxonomy. Second, we compared the concepts in the Body Listening and Self-Management Taxonomy with concepts that were automatically identified from an extant health knowledge resource, the Unified Medical Language System (UMLS), to better characterize the information that participants contributed. Third, we employed visualization techniques to explore the concept space of the tags. A correlation matrix, based on the extent to which categories tended to be assigned to the same tags, was used to study the interrelatedness of the taxonomy categories. Then a network visualization was used to investigate structural relationships among the categories in the taxonomy.

**Results:**

First, we proposed a taxonomy called the Body Listening and Self-Management Taxonomy, with four meta-level categories: (1) health management strategies, (2) concepts and states, (3) influencers, and (4) health-related information behavior. This taxonomy could inform future efforts to organize knowledge and content of this subject matter. Second, we compared the categories from this taxonomy with the UMLS concepts that were identified. Though the UMLS offers benefits such as speed and breadth of coverage, the Body Listening and Self-Management Taxonomy is more consumer-centric. Third, the correlation matrix and network visualization demonstrated that there are natural areas of ambiguity and semantic relatedness in the meanings of the concepts in the Body Listening and Self-Management Taxonomy. Use of these visualizations can be helpful in practice settings, to help library and information science practitioners understand and resolve potential challenges in classification; in research, to characterize the structure of the conceptual space of health management; and in the development of consumer-centric health information retrieval systems.

**Conclusions:**

A participatory platform can be employed to collect data concerning patient experiences of health management, which can in turn be used to develop new health knowledge resources or augment existing ones, as well as be incorporated into consumer-centric health information systems.

## Introduction

### Background

In recent years, there has been increased interest in Web-based platforms that aim to derive value from user participation through crowdsourcing, collaborative, and participatory frameworks. Efforts to leverage “collective intelligence” include the collective authoring of Wikipedia content, shared tagging of photos on Flickr, sharing of bookmarks on Del.icio.us, and collective annotation of museum artifacts [1-5]. Collaborative tagging, a practice in which users add meta-data to shared content, can be useful when personnel are not readily available to perform classification tasks [6], as a channel for nonprofessional catalogers to participate in meta-data creation [7], and as an approach to organize knowledge by users’ own language [8].

Due to their collaborative and ad hoc nature, tagging systems inherently lack the essential properties characterizing controlled vocabularies, and low precision and lack of collocation are common issues [9]. However, this very nature also provides support for multiplicity of perspectives, collective interpretation, sense-making, and meaning production; and promotion of a collaborative, democratic, and participatory style of knowledge construction and organization [10-12].

Collaborative tagging presents a special opportunity in biomedical knowledge organization. There are numerous knowledge resources such as the Unified Medical Language System (UMLS) Metathesaurus [13] and the Systematized Nomenclature of Medicine-Clinical Terms (SNOMED-CT) [14], which facilitate inference in biomedical and clinical domains. However, there has been increased awareness that health consumers possess a type of expertise that is different from that of clinicians and that user-generated content can be a valuable source of health knowledge [15]. As such, there has been work that has employed social media data to improve existing knowledge resources, including the use of PatientsLikeMe data to augment the open access and collaborative Consumer Health Vocabulary [16] and the use of community-generated text to map professional medical terms to their consumer equivalents [17]. Other research has investigated the overlap between social media data sources such as PatientsLikeMe and YouTube and SNOMED-CT [18,19]. It has also been shown that search log data share similarities to folksonomy tags and can be used to improve controlled vocabularies and information retrieval [20].

The use of social media data to augment professional controlled vocabularies is important work. In this study, we concentrated our efforts in a different direction: to employ collective intelligence to develop a knowledge resource focused on patients’ health management strategies. To set the context for the knowledge resource that we aimed to build, we now review extant literature on self-management of chronic illness, body listening, and related terminology.

### Self-Management of Chronic Illness and the Importance of Body Listening

Over the course of a chronic illness, people learn to manage their health in different ways. Self-management of chronic illness has been characterized as a dynamic and daily experience involving three main categories of processes: focusing on illness needs, activating resources, and living with a chronic illness [21]. It has been argued that though there is considerable extant research on self-management barriers and facilitators, the developmental patterns and sustainability of self-management over time remain largely unknown [22]. In this study, we set out to investigate how people form and acquire self-management skills, particularly those relating to body listening and body awareness.

Body awareness, defined as the ability to recognize subtle body cues, can be helpful in the management of many conditions including chronic low back pain, congestive heart failure, chronic renal failure, and irritable bowel syndrome [23]. In the context of fibromyalgia, patients have reported learning over time what their pain triggers were, coming to understand what foods they were sensitive to, and when they had hit their limit and needed to rest [24].

Combining an awareness of embodied experience with information from test results, that is, combining one’s own knowledge with a biomedical understanding of a condition, has also been referred to as “knowing one’s body” [25]. Body listening has also been described as the “subprocesses of physical self-assessment and applying a personal filter through which to interpret that information” [26] (p. 265).

Body awareness and body listening also share similarities with concepts such as self-awareness and self-monitoring. For example, previous research has investigated the self-awareness of the cues, sensations, and circumstances that people with diabetes associate with hypoglycemia, euglycemia, and hyperglycemia, and the types of strategies that they used to tune in to these body cues [27]. Self-monitoring has been defined as having two components: (1) awareness of bodily symptoms, sensations, daily activities, and cognitive processes; and (2) measurements, recordings, and observations that inform cognition or provide information for independent action or consultation with care providers [28].

The importance of attending to body cues in health management suggests that a greater understanding of the ways in which people engage in these activities is warranted. Moreover, patients and clinicians may have different ways of describing their symptom experiences [29]. Thus, if the knowledge that patients acquire over time could be effectively captured, this information might be incorporated into health knowledge resources and shared on a wider scale. To take a step to fill this need, we developed a platform where people could post their experiences concerning body listening and how they learned or were learning to do it, in the hope that the data collected could later be used to develop a system for organizing and exploring knowledge relating to body listening.

### Research Questions

This study was based on a premise of the value of coconstruction of knowledge. We developed a platform on which project team members and study participants could collectively engage in discussion on topics relating to body listening and, more broadly, health management. We targeted chronic conditions requiring self-care in which patients might share symptomatology, with a particular focus on chronic pain. These conditions included chronic pain, fibromyalgia, arthritis, and multiple sclerosis and common comorbidities such as chronic fatigue syndrome and irritable bowel syndrome.

Over the course of 10 weeks, participants engaged in a moderated discussion of topics relating to body listening. As they engaged in discussion, participants were also encouraged to provide tags that described the content that they shared. This article investigates whether asking participants to provide tags can add value to the data that they contribute and what the potential implications of this value might be to knowledge organization. We investigated three main research questions:

RQ1. What types of subject matter were represented in the tags?

RQ2. How does concept coverage in the Body Listening and Self-Management Taxonomy, proposed in RQ1, compare with that of the UMLS Metathesaurus?

RQ3. To what extent were Body Listening and Self-Management Taxonomy categories assigned to the same tag, and what does this pattern of category cooccurrences suggest about the relationship between categories?

## Methods

### Data Collection

#### Study Platform and Content Development

**Figure 1:**
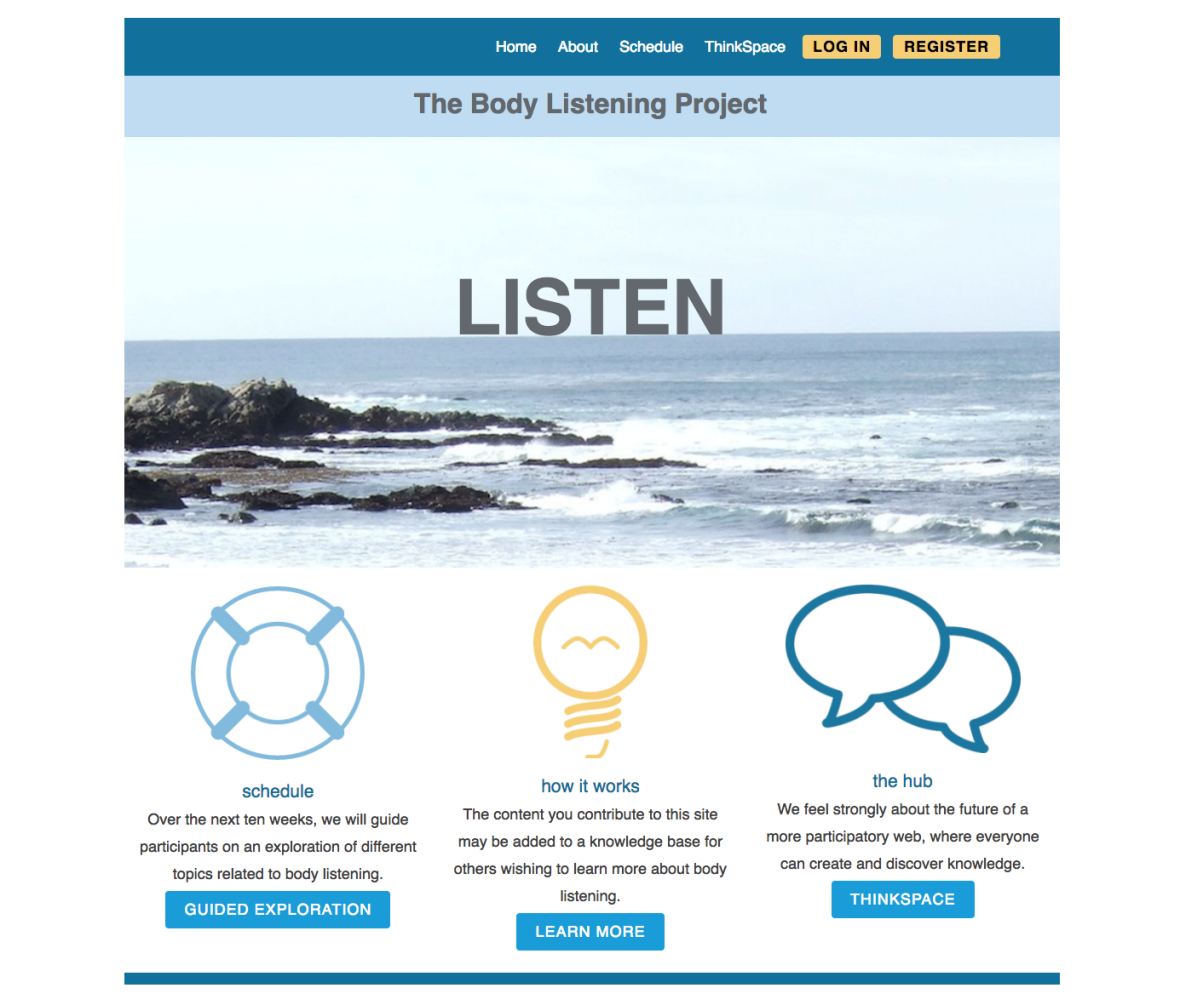
The Body Listening Project index page.

This study involved a 10-week “Guided Exploration” in a discussion forum style space built using the Wordpress platform (Figure 1). The discussion forum itself was called the “ThinkSpace,” to emphasize the value of participants’ contributions. Data collection took place over the course of a 10-week period involving moderator-facilitated discussions of topics relating to body listening. Each week, a new theme was introduced, and each day, a new question relating to the theme was posted (Table 1).

**Table 1:** Guided Exploration schedule.

Week no.	Topic
1	Getting in touch with your body rhythms
2	Movement, energy, and fatigue
3	Food and environment
4	Pain management
5	Mood management
6	Sense-making and conveying what your body tells you in health care contexts
7	Conveying what your body tells you in life contexts
8	Tuning in to your body with arts-based techniques
9	Mindfulness as a way to get in touch with your body
10	The body as a vehicle for self-growth

Though there are processes that may be shared between individuals who engage in body listening and self-management, the set of experiences and perspectives is admittedly diverse. It was necessary to limit the topics covered so that they could be explored in the 10-week study period. The themes employed an experiential line of inquiry, and the content was tailored toward chronic conditions requiring self-care that overlapped in symptomatology, with chronic pain as the primary characteristic.

Selection of topics was based on a two-step process. At the outset, the first author developed a list of seed topics based on three sets of research literature: (1) self-management of chronic illness; (2) body listening and body awareness; and (3) fibromyalgia, arthritis, multiple sclerosis, and other chronic pain and rheumatological conditions. The moderators subsequently discussed these topics, added their own, and then finalized the set of topics to explore in the Guided Exploration. The content was collectively developed by the team through a series of biweekly meetings and the collaborative authoring of a shared document.

Moderators monitored the site regularly to engage the group and to respond to posts by site participants. Participants were encouraged to author posts and then add one or more tags after the main body of content. Participants were informed that the experiences they shared in the ThinkSpace would be considered data, and they gave consent as part of the account creation process. The study procedures were approved by the Institutional Review Board of the University of Washington School of Medicine.

The team comprised 10 people: a project manager, faculty advisors, moderators, a site administrator, and communications and outreach personnel. Members came from multiple disciplines including library and information science, biomedical informatics, nursing, and public health. Many team members also had experience with chronic illnesses.

#### Sample and Study Recruitment

The overall goal of this study was to increase our knowledge of body listening and body awareness, which can potentially be helpful in the management of many chronic conditions [23]. The study was open to anyone over the age of 21 years who was interested in, or wanted to contribute to, knowledge on the phenomenon of “body listening.” We employed two types of recruitment strategies. First, we employed recruitment strategies that were not focused on a given disease or condition, such as university and hospital listservs and posted flyers in high traffic areas such as university campuses and coffee shops.

Second, we recruited participants through multiple social media channels including Facebook, Reddit, Twitter, and health discussion forums. Our social media recruitment focused on particular groups of interest including groups focused on chronic pain, fibromyalgia, and discussion groups for commonly occurring comorbidities such as chronic fatigue syndrome and irritable bowel syndrome. We also reached out to people of different demographic characteristics including ethnicity and education level.

### Data Analysis

#### RQ1. What Types of Subject Matter Were Represented in the Tags?

To analyze the tags that were collected, the posts were exported from the MySQL database where they were stored, and a PHP script was used to extract all of the tags. A hashtag was defined as a word or set of words strung together without spaces, preceded by a hash or pound sign (#). Hashtags can be useful for assisting users to find content of interest. There were some malformed tags—cases in which multi-word tags included spaces, such as “#cell phone alarm,” and instances in which tags started and ended with pound signs, such as “#meditation/prayer time#.” Twelve such errors were caught through manual lookup, and the tags’ formats were corrected for inclusion in the analysis. We did not correct for spelling errors and spelling variants, as we believed that this would be useful information to preserve.

Though it was expected that many participants might have familiarity with formatting guidelines for social media and microblog hashtagging, we developed an informational page that explained to participants how to author content. A link to this “Contribution How-To” page was available from the main ThinkSpace page. Sample tags were provided to demonstrate tag content and formatting. However, to avoid limiting the participants’ conceptualization of what a tag was, an explicit definition of a tag was not included as part of this page.

To understand the nature of the tags that were used, the tags were manually assigned to one or more categories by the first two authors, and a relational hierarchy of the codes developed, as follows. First, the two coders independently assigned categories to a subset of the data (n=100). They discussed these codes and categories until they reached agreement about the categories and the hierarchical relationship between them. Then they coded a new subset (n=100) and calculated three different types of inter-rater reliability (IRR). A more extensive discussion of the types of IRR used appears in a prior work [30], but a summary of the types of IRR appears in Multimedia Appendix 1. In this round of coding, they achieved a high level of agreement, so the second author proceeded to code the rest of the tags. The third author also took part in the decision-making process concerning the relationships between the categories.

This taxonomy was later slightly refined to improve the clarity of category definitions, as well as to increase the inclusiveness and logical consistency of the framework by renaming the meta-category, “gathering and conveying information,” to “health-related information behavior,” and adding the “health-related information behavior” category. All three authors took part in the discussion to refine the taxonomy. The taxonomy is presented in the Results section.

There were situations in which the meanings of the tags were unclear. For example, #isolation may refer to a state, a treatment modality, or exercise. Ambiguous tags were analyzed and categorized within the context of the posts in which they occurred. A list of the tags, along with their classifications, appears in Multimedia Appendix 2. If a tag was not formatted correctly in the original contribution, the original malformed tag appears in parentheses.

#### RQ2. How Does Concept Coverage in the Body Listening and Self-Management Taxonomy Compare With That of the Unified Medical Language System (UMLS) Metathesaurus?

To investigate the extent to which tags expressed concepts covered in the UMLS Metathesaurus, we manually separated all multi-word tags into their individual word components and used the MetaMap API to identify relevant UMLS concepts in these tags [31]. We then performed error analysis of a subset of the tags for which MetaMap identified relevant concepts (n=200) to evaluate the degree to which the identified concepts captured the meaning of the specified tags. Identified concepts were considered “correct” if they captured the essence of the meaning of the corresponding tags, “incorrect” if the meaning of the identified concepts did not match the meaning of the tag, and “incomplete” if there were critical parts of the meaning that were not captured. We also identified the sources of error if an identified concept was labeled “incorrect” or “incomplete.”

To visually compare the concept coverage, we generated category frequency distributions for both the Body Listening and Self-Management Taxonomy and for the UMLS using Python.

#### RQ3. To What Extent Were Body Listening and Self-Management Taxonomy Categories Assigned to the Same Tag, and What Does This Pattern of Category Cooccurrences Suggest About the Relationship Between Categories?

In this research question, we employ visualizations to explore the concept space of the contributed tags. A correlation matrix, based on the extent to which categories tended to be assigned to the same tags, was used to study the interrelatedness of the taxonomy categories. Then, a network visualization was used to explore the concept space of the tags contributed and investigate the structural relationships among the categories in the taxonomy.

To examine conceptual overlap, we employed the multiple tag assignments to visualize the correlations between categories based on their tendencies to be assigned to the same tags. First, the tags and their category assignments were used to construct an N × N cooccurrence matrix based on the frequency at which categories were assigned the same tag and categories were considered to cooccur with themselves. This matrix was then converted and visualized as a diagonal correlation matrix using Python.

We also investigated how strongly categories were related to one another, both within and across meta-categories, as well as their overall prevalence within the tag corpus using a network visualization. Network analysis is used in numerous disciplines and has been used to analyze many naturally occurring phenomena including communication patterns in an emergency department [32], the spread of disease outbreaks [33], and the structure of research in a given subject area or discipline [34]. Other uses of network analysis include the analysis of structural relationships among entities [35] and identification of community structure [36]. Network structures can also be used to examine the organization of human semantic knowledge [37]. In this study, we employ it to examine the relationships between the categories in the Body Listening and Self-Management Taxonomy.

The open graph visualization software Gephi was used to visualize the network of tags [38]. We rendered a network in which each node represented a category in the taxonomy. The size of the node was based on the number of times that the category was assigned to a tag. The nodes were assigned colors based on their meta-categories, such that all nodes of the same meta-category shared the same color. Two nodes were connected if they were ever assigned to the same tag within the corpus, and the weight of the edges was determined by number of times they were assigned to the same tag. The ForceAtlas2 layout was used to visualize the network [39] and the Label Adjust algorithm to eliminate visual overlap in labels.

## Results

### Data Collection

Over the course of the 10-week study, 234 participants registered to participate. As expected, the range of conditions that participants reported was diverse, with greater emphasis on pain conditions, mental health conditions, food sensitivities, irritable bowel syndrome, chronic fatigue syndrome, and thyroid disorders—all areas which were emphasized in the social media recruitment. A total of 28 participants posted in the discussion forum. This participation pattern is consistent with prior research on discussion forums, in which those who participate are a fraction of the countless others that may be “lurking” [40].

Altogether, participants and moderators authored 431 posts and used 818 tags. Of these, 680 tags were unique, and the tags used more than once were used 2-6 times. Some tags such as emotional freedom technique (EFT) and traditional Chinese medicine (TCM) appeared both in their traditional and acronym forms. Approximately a quarter of the posts did not contain tags (n=114), and many posts that included tags featured multiple tags (n=197).

Though many tags were single words (n=163), others were combinations of words (n=517). Many expressed a concept, including #attitudeiseverything, #taketimetounderstand, and #nothavingmypaininadvance. This last tag expressed the concept of trying to be positive and dealing with situations as they occur, rather than worrying about pain that one might experience later. There were instances in which tags were assigned to multiple categories such as #nightpain, which was classified as symptoms and illnesses (IS), rhythms and schedules (RS), and influencers (IF).

### Data Analysis

#### RQ1. What Types of Subject Matter Were Represented in the Tags?

The contributed tags were grouped into thematic categories and an initial relational structure for the categories developed, validated, and reported in prior work [30]. The validation procedure is summarized in Multimedia Appendix 1. In this research question, we focus on describing the proposed taxonomy and exploring its implications for the classification of subject matter concerning health management. There were four meta-level categories: health management strategies, concepts and states, influencers, and health-related information behavior (Table 2).

##### Health Management Strategies

This meta-category encompasses the wide variety of strategies that people may employ to manage their health. In addition to treatments (TM), people may utilize other strategies including exercise (E), diet and food (DF), coping or coping strategies (CS), and supplies and equipment (SE). Supplies and equipment was classified under health management strategies because participants would share examples of tools that they used to manage their health, such as #netipotforallergies. Strategies that did not clearly fall into any of the other categories were assigned the category health management strategy (HMS). Examples of this include #cellphonealarm and #taketimetorecover.

##### Concepts and States

Concepts and states comprised three categories: general concepts (CN), positive actions (PA), and mental states (MS). Certain concepts had an inherently positive orientation, and these were classified as positive actions. These included terms such as #advocatingforyourself and #transformation. There were others that referred to mental states, such as #acceptance, #compassion, and #nofear. Finally, concepts that were more generic, such as #journey, #balance, and #energy were simply classified as general concepts.

##### Influencers

Though one may not always be aware of factors that affect their body condition, nevertheless there are factors that influence the state of one’s body at any given point in time. The meta-category, influencers, was used to describe these factors and included two categories: outside factors influencing the body and mind (IF) and body rhythms and schedules (RS). Examples of the former included seasonal influences, such as #fourseasons and #pollenispainful, and examples of the latter included internal factors, such as #nightowl and #mostproductivetimes.

**Table 2:** Body Listening and Self-Management Taxonomy.

Meta-category			Description	Examples
	Code	Category		
**Health management strategies**				
	HMS	Health management strategy	Health management strategy	Cellphonealarm, taketimetorecover, fulltimebodymanager
	TM	Treatment or treatment strategy	Treatments (physical, psychological, other) for managing chronic illness	acupuncture, alexandertechnique, biofeedback, funkplunkmyselfoutside
	E	Exercise	Exercises and movements, as part of a treatment or not	Adductorstretch, walking, yamunabodyrolling, exercisewithchildren
	DF	Diet and food	Food, herb, and supplement consumption practices and principles	atkinsdiet, eliminationdiet(s)
	CS	Coping or coping strategies	Strategies for coping with pain or illness	prayer, affirmations, reframing
	SE	Supplies or equipment	Tools or equipment for tracking, treating, or coping with chronic pain or illness	Netipotforallergies, bodypillow
**Concepts**				
	CN	General concepts	General concepts relating to body listening	Attitudeiseverything, journey, balance, energy
	PA	Positive actions	Actions with a positive outlook or orientation	advocatingforyourself, transformation, pushingthrough
	MS	Mental states	Mindsets and approaches to manage life with pain or illness	acceptance, compassion, nofear
**Influencers**				
	IF	Influencers	Outside factors influencing the body or mind	Sickweatherchanges, fourseasons, hotweather, pollenispainful
	RS	Rhythms and schedules	Rhythms around and of the body	Nightowl, mostproductivetimes
**Health-related information behavior**				
	HIB	Health-related information behaviors	Health-related information behavior such as seeking and/or sharing information	findinghealthinformation
	HR	Health-related resources	Resources for gathering health-related information	Patientexperts, trustedsites
	SM	Self-monitoring	Becoming aware of body rhythms, treatment, or symptoms	Journaling, symptomtracker, sleepdigestionconnection, bodyclues
**Other categories**				
	IS	Symptoms, illnesses	Symptoms, illnesses, or diseases	Shakiness, sleepdisorders
	HC	Health care-related terms	Health care-related terms	doctors, massagetherapists, physicaltherapist
	CM	Communication and relationships	Interactions, communications, and relationships with others and the self	Energyfromothers, supportivebossesrock
	AD	Moderator or administrative content	Moderator instructions	Useanycoloryoulike, usebothhands

##### Health-Related Information Behavior

This meta-category comprised three categories, health-related information behaviors (HIB), health-related resources (HR), and self-monitoring (SM). Examples of health-related sources of information that participants mentioned include #patientexperts and #trustedsites. The category of self-monitoring included both monitoring that included devices, as well as self-monitoring and exercising awareness without devices, such as #bodyclues.

Health-related information behaviors was used to classify all other terms that fell under this meta-category but not specifically under any of the other two terms. In this study, these terms were extremely rare, but we believe that health-related information behaviors is an important category and that it could be further differentiated into additional categories. Thus, we have chosen to retain it as part of the framework.

It may be useful to consider the relationship between the two meta-categories, health-related information behavior and health management strategies. Health-related information can be used to manage one’s health, and in the context of this study, often was. However, having information as a separate meta-category was intended to emphasize that in these activities information played the central role and health was the supporting context. For example, the tag #findinghealthinformation is primarily about information and secondarily about health. In situations in which both meanings were equally salient, a tag might be categorized under each meta-category. One example was #journaling, which was categorized as both self-monitoring (SM) and treatment or treatment strategy (TM).

##### Other

An additional set of categories did not fit under any meta-level category. These include symptoms and illnesses (IS), health care-related terms (HC), communication and relationships (CM), and moderator or administrative content (AD). Symptoms and illnesses help us to understand how participants view their own health, and health care-related terms and communication and relationships help us to understand elements of a person’s context that play important roles in their long-term health management. The last category, moderator or administrative content, was most prominent in Week 8: Tuning in to Your Body with Arts-Based Techniques, in which the moderator provided instructions for participants to explore and express their physiological sensations using arts-based techniques.

#### RQ2. How Does Concept Coverage in the Body Listening and Self-Management Taxonomy Compare With That of the UMLS Metathesaurus?

**Figure 2:**
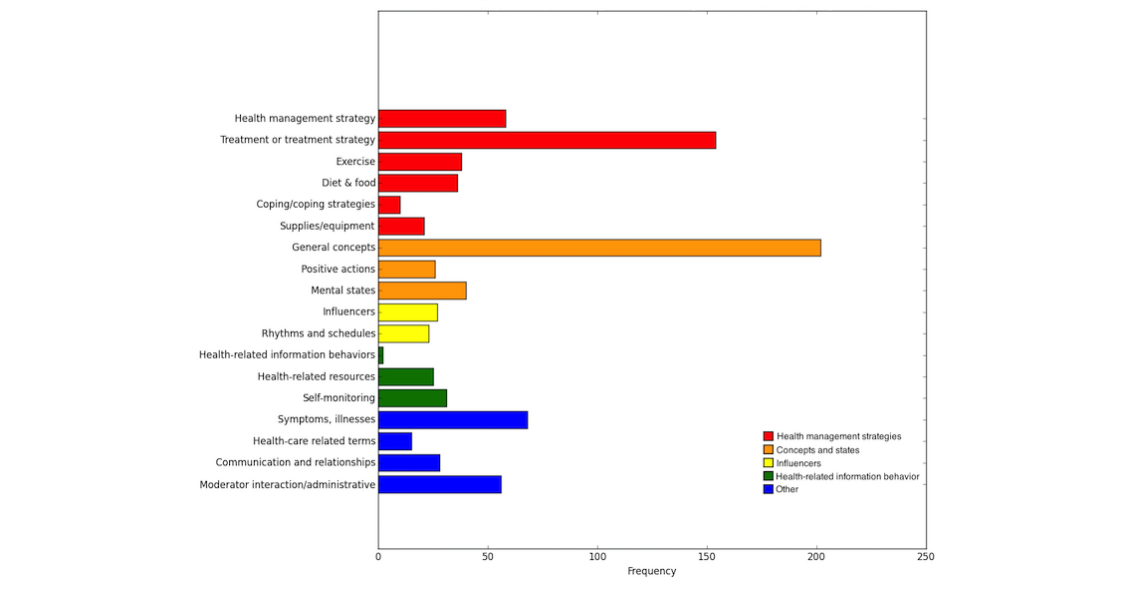
Concept coverage in the Body Listening and Self-Management Taxonomy.

**Figure 3:**
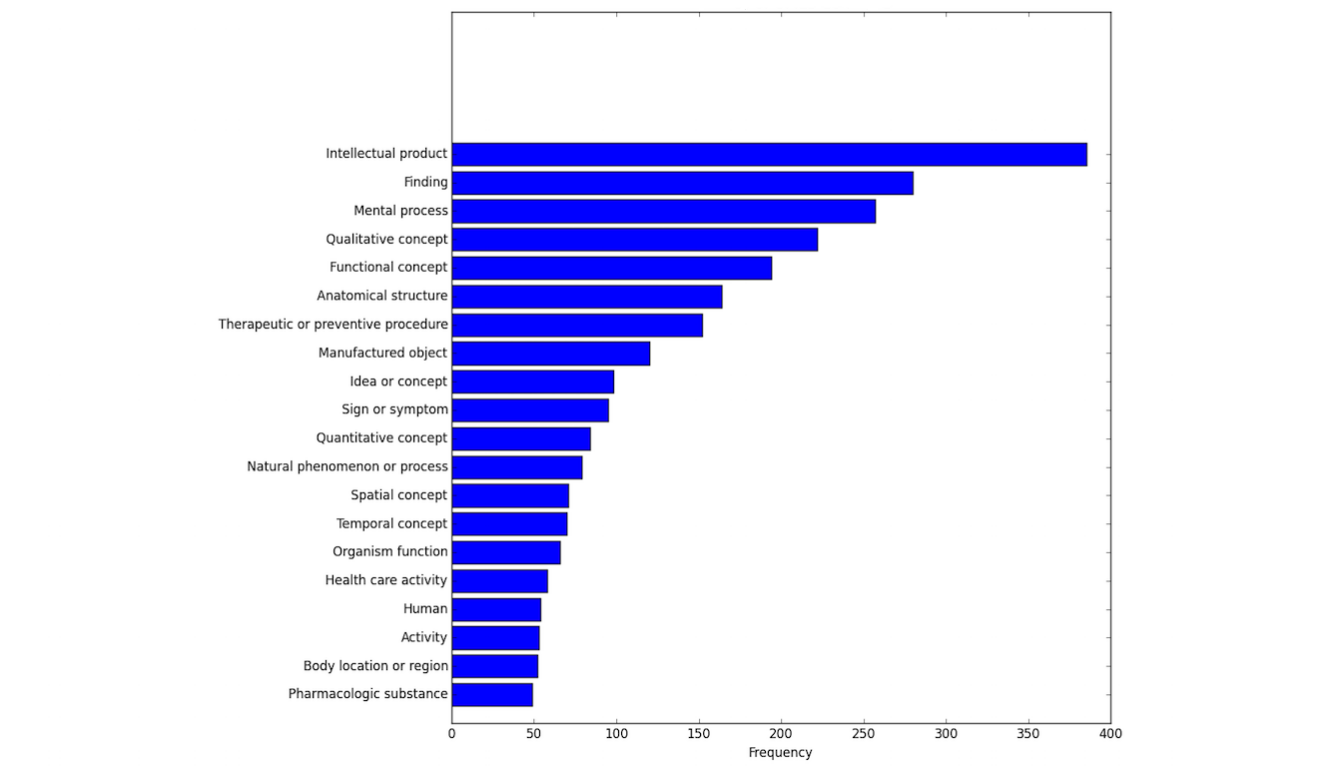
Concept coverage in the UMLS (Unified Medical Language System) Metathesaurus.

In this section, we compare the prevalence of category assignments based on the Body Listening and Self-Management Taxonomy (Figure 2) with the top 20 UMLS categories that were automatically identified using the Metamap API (Figure 3). The category prevalence includes multiple category assignments, meaning that for tags that were assigned to multiple categories in the Body Listening and Self-Management Taxonomy or in the UMLS, each category assignment is depicted in the frequency distributions.

Examining the prevalence of categories across the tag corpus can help us to identify subject matter areas in which the study was successful in eliciting information. In so doing, we may also identify important factors for people to consider as they address their health management needs. In the Body Listening and Self-Management Taxonomy, health management strategies and more specifically, treatments, are one area in which participants contributed a great deal of content. A variety of treatments were mentioned, including widely known treatments such as #acupuncture and #biofeedback, lesser known techniques such as #alexandertechnique, and more original references such as #funkplunkmyselfoutside. Whether health management strategies are generally well-known or not, it can be useful to have them aggregated in one place. The more original ones may “speak to” or resonate with participants in a way that traditional resources may not.

Another area in which there was extensive coverage was the category of general concepts. Though this category sounds fairly broad, we believe that additional study of the emergent concepts can lead to increased knowledge of important areas of self-management. The UMLS category of intellectual product perhaps roughly corresponds to the category of general concepts in the Body Listening and Self-Management Taxonomy.

We also investigated the extent to which concepts reflected in the tags were covered in the UMLS Metathesaurus. Altogether, 101 concepts were extracted; we show the highest frequency concepts in Figure 2. Some of the identified concepts are fairly clear in terms of their meaning, for example, sign or symptom and pharmacologic substance. Many, though, are too general (eg, “functional concept” or “qualitative concept”) to be useful to a health consumer searching for information.

The MetaMap API did not identify concepts for 11% (74/680) of the terms. We performed error analysis for a subset of the tags for which MetaMap identified UMLS concepts (n=200). Among these, MetaMap correctly identified one or more high-level classifications of the tags 60% (120/200) of the time, incorrectly identified the meaning 15.5% (31/200) of the time, and provided an incomplete set of relevant concepts 24.5% (49/200) of the time. Additionally, there were situations in which MetaMap correctly identified relevant concepts but produced noise through the identification of extraneous concepts. The primary sources of error (for incomplete or incorrect meaning) are shown in Table 3.

**Table 3:** Primary sources of error in automatic tag classification via the MetaMap API.

Issue	Example tag	Prevalence (%)
Not a strictly health-related concept	#supportivebossesrock	15
Meaning of an identified word is incorrect	#spiders (refers to the sensation on the skin, not the eukaryote)	12
Missing a concept	#noscentedcandles (missed “no” and “candle”)	11
Missing an interrogative word	#whatthebodywantstoeat	6
Missing a verb	#dowhatworksforyou	4
Is a health-related concept but is not included	#structuralintegration	4
Missing mental process	#distancethepain	3

Overall, automatic identification worked better for tags comprising one or two words. The more words that were involved, the more the tags tended to express a concept that was not well represented in its entirety by the identified concepts. Based on the error analysis in Table 3, areas for improvement of the UMLS include the integration of additional terms, including emergent terms such as structural integration; alternative and colloquial senses of words such as spiders on the skin; and contextual aspects of health management, such as workplace wellness.

These results suggest that though extant health knowledge resources might be used to characterize consumer-generated hashtags, additional work is necessary to support health consumers’ information-seeking. Though this is a recognized problem that has received attention [16,41], there is still much that we can do to improve the UMLS coverage in terms of colloquial and patient or consumer-oriented language. Moreover, the results of this study suggest that, besides the addition of consumer-centric terms, there is also a need to add concepts to the UMLS. Lastly, the Body Listening and Self-Management Taxonomy could potentially fill an important gap in terms of providing conceptual categories that reflect patients’ self-management strategies.

#### RQ3. To What Extent Were Body Listening and Self-Management Taxonomy Categories Assigned to the Same Tag, and What Does This Pattern of Category Cooccurrences Suggest About the Relationship Between Categories?

There were instances in which contributed tags were assigned to multiple categories. A total of 513 tags were assigned to only one category, and 167 tags received multiple assignments. In this section, we investigate the extent to which categories tended to be assigned to the same tag. The instances of multiple category assignment can help us to understand the naturally occurring relationships between taxonomy categories, the extent of their semantic overlap, and most importantly for practice, the extent of ambiguity that may be present during a manual process of classification.

**Figure 4:**
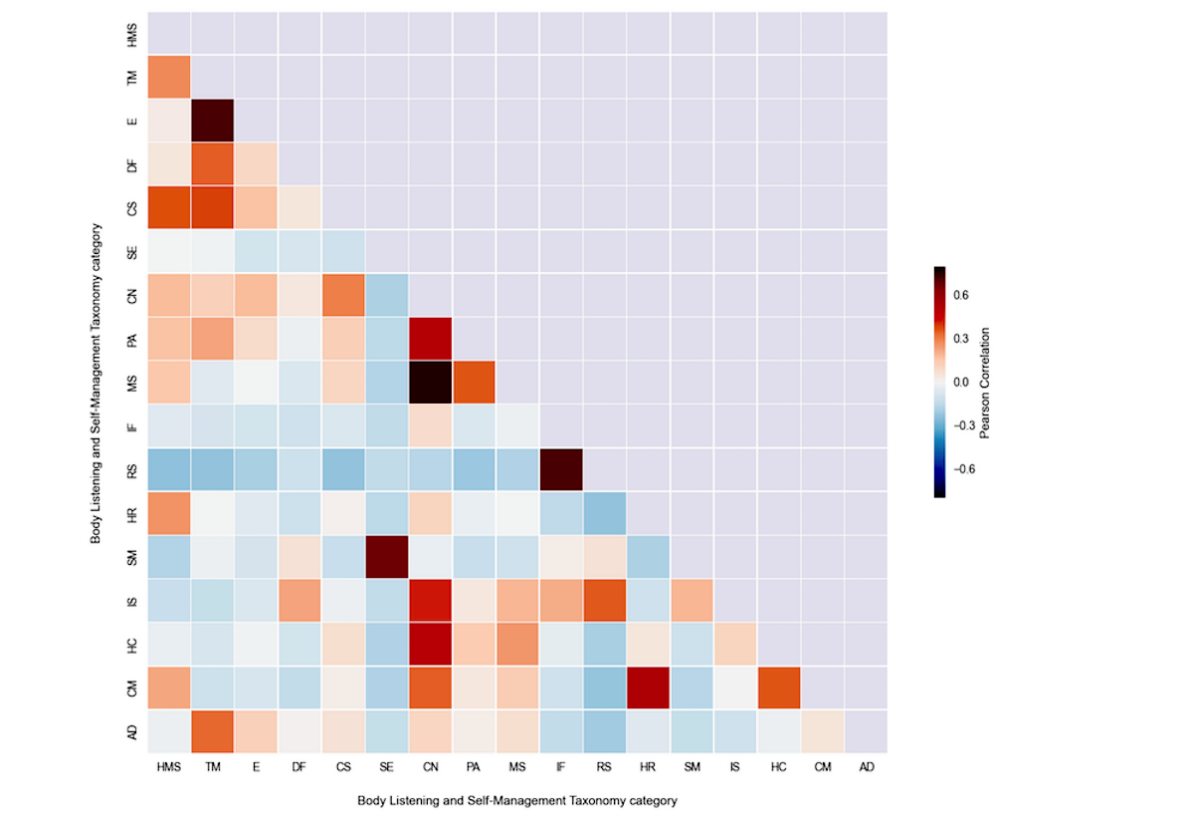
Co-occurrence of categories in tag classification.

We generated a diagonal correlation matrix to examine the tendency for categories to be assigned to the same tag (Figure 4). In this matrix, category combinations with a higher correlation, that is, a greater tendency to cooccur, are denoted in red. Health-related information behavior did not cooccur with any other categories during category assignment and thus, does not appear in the matrix.

Multi-category assignments were common among the health management strategies categories and among the three concepts and states categories. Examples of the former type of multi-category assignment include #hellerwork and #bodyrolling, both assigned to the exercise as well as treatment or treatment strategies category. Examples of overlap in the concepts and states categories include #compassion (CN, PA, and MS) and #letitgo (CA and PA). The pattern of correlations also reflects our own experiences in category assignment, in which we occasionally found it difficult to decide between treatment categories and concept categories because of the multiple senses that might be conveyed in tags. The cooccurrence of influencers with rhythms and schedules was also common. Examples of these include #fourseasons and #hotweather.

Another interesting aspect of these cooccurrences is the moderate correlations between the concepts and states categories and various categories within the health anagement strategies meta-category. The tags, #advocate4yourself (PA, HMS, and CN), which referred to advocating for oneself in workplace environments, and #allowingsadness (CN, PA, and TM), illustrate the importance of concepts and positive attitude in health management.

**Figure 5:**
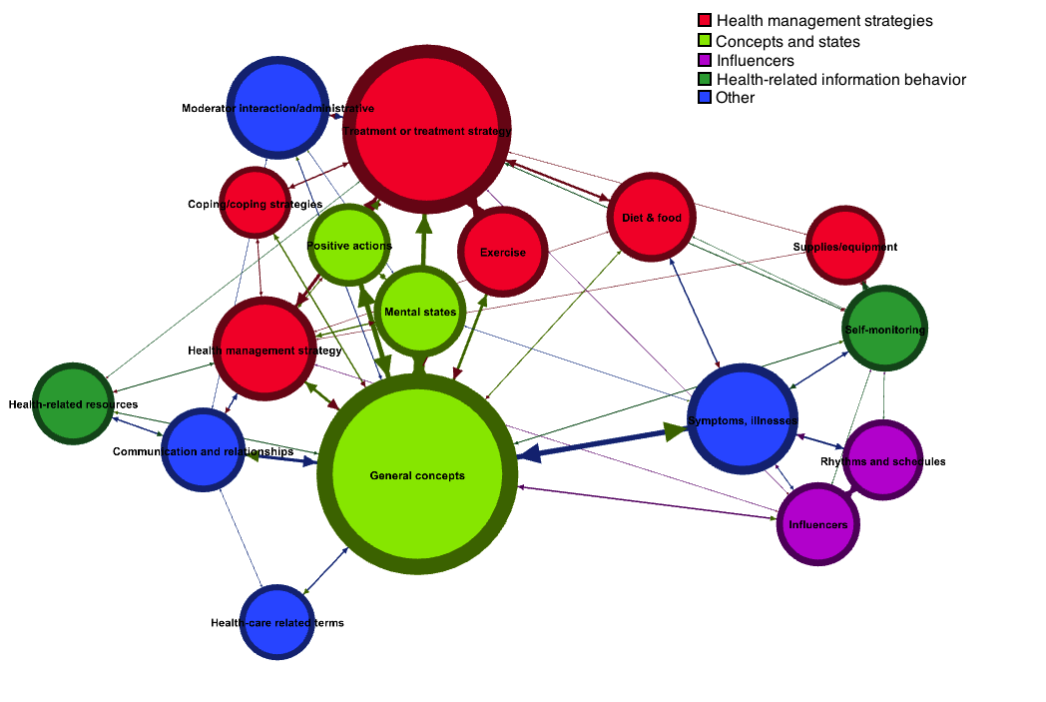
Body Listening and Self-Management Taxonomy as a network.

We also investigated the structural relationships between the categories—how strongly they were related to one another, both within and across meta-categories, as well as their overall prevalence within the tag corpus. We rendered a graph visualization as described in the Method section (Figure 5) and used the resulting visualization to engage in an interrogative dialogue concerning the relationships between the categories of the Body Listening and Self-Management Taxonomy. The larger the nodes, the greater their prevalence in the tag corpus, and the closer the category nodes were to each other in the network, the more times that they were assigned to the same tags. Nodes of the same color belonged to the same meta-category. As before, the health-related information behavior category does not appear because it did not cooccur with any other categories during category assignment.

The resulting visualization illuminates some interesting relationships between the categories. For example, most categories appear closer to other categories within the same meta-category. Categories under health-related information behavior are a notable exception. Though these three categories share conceptual similarity in that they all relate to how people interact with information, in the case of self-monitoring, there appeared to be a stronger conceptual connection with devices (supplies/equipment). Among the categories that were not subsumed under a meta-category, the position of symptoms and illnesses is particularly interesting. The proximity of the symptoms and illnesses node to rhythms and schedules underlines the importance of rhythms and temporal associations to symptoms. Examples of tags which were assigned to both categories include #reverseSAD, #daysnightsreversed, and #migraineuponwaking.

The proximity of categories to one another can provide insight into factors that may be important to consider together in body listening and health management. For example, this network shows that communication with others, mental states and health management strategies can be closely related. Whereas this may seem obvious, it provides further evidence that, in working with patients, it can be helpful for health care practitioners to consider contextual factors such as the patients’ support network and mental state in assisting them to select, adopt and maintain health management strategies. The interrelatedness of the categories, as depicted in the figure, highlight the importance of not treating any aspect of a patient’s care as if it occurs in a vacuum.

## Discussion

### Principal Contributions

This paper describes the development of a participatory platform for people to contribute their experiences of body listening and presents an analysis of the tags collected. This analysis makes several original contributions. First, we proposed a preliminary classification scheme, the Body Listening and Self-Management Taxonomy, for concepts associated with body listening. Second, we compared the concepts in the Body Listening and Self-Management Taxonomy with the concepts from the UMLS Metathesaurus that were automatically identified from the tags. This comparison served to characterize the information that patients contributed through the participatory platform and demonstrated that the tags offer important information concerning health management, particularly with regard to self-management strategies and contextual factors affecting health, such as seasonal and body rhythms, mental state, communication, and interpersonal relationships. Additionally, the comparison showed that the taxonomy could be used to classify patients’ health management strategies more descriptively than could be achieved using UMLS concepts. Third, we examined the extent to which tags were assigned to multiple categories of the Body Listening and Self-Management Taxonomy, to gain insight into how the categories might be related to one another. Though the categories in any given meta-category generally appeared together, there were key areas of connectivity between the categories and meta-categories. Identifying these connections can help us to form a greater understanding of the need to consider factors affecting our health in context.

### Considering the Value of the Data Collected Through the ThinkSpace

This study was intended as a starting point for building a public resource on body listening, and more broadly, self-management of chronic conditions. We used an approach combining user contribution of tags with subsequent curation to develop a taxonomy for use in the development of future knowledge resources.

This taxonomy is a preliminary step to improve the classification and retrieval of content that is presented to health consumers. We employ a two-step process that offers health consumers the opportunity for greater involvement by enabling them to not only provide data but also influence how it is organized through the tags that they provide. However, folksonomies come with certain issues, such as a preponderance of terms with different levels of specificity. Thus, we added an additional layer of human curation and classification to create a schema that is emergent from the data, and thus, more consumer-centric.

One important implication from the study results concerns the subject matter content of the contributed tags and the manner in which this data might be utilized. The ThinkSpace resulted in tags that focused on health management strategies, concepts, and contextual information about people’s health experiences. These tags provide insight into the actions that patients take to manage their health, the realities that they face as they address their health issues, and the resources that they have access to, from their perspectives. Thus, the tags might be incorporated into a system that patients, caregivers, and health consumers in general can use to find information about their health needs and interests.

Though systems that provide health information to consumers do exist, they are generally focused around traditional medical concepts. For example, the Medline Plus interface currently includes section headings and subheadings such as “Health topics: find information on health, wellness, disorders, and conditions,” “Drugs and supplements: learn about prescription drugs, over-the-counter medicines, herbs, and supplements,” and “Medical encyclopedia: articles and images for diseases, symptoms, tests, treatments.” Incorporating tags used in the ThinkSpace, such as #funkplunkmyselfoutside, #exercisewithchildren, and #supportivebossesrock, or the concepts expressed therein, may resonate with health information seekers as well as serve as an alternative channel through which to access information. In addition, our findings suggest that meta-data such as body rhythms, external influences, concepts, and attitudes could potentially serve as entry points for finding information. Concepts in the rhythms and schedules category, such as having pain at night and difficulties due to hot weather may resonate with patients but are less likely to appear as indices or headings in traditional consumer health information systems. Yet they are nevertheless important to health management. Thus, user-contributed tags could be incorporated into health information retrieval systems to make them more user-friendly and intuitive.

### Facilitating Tag Classification and Taxonomy Enrichment

In this study, we classified the tags using two methods: manual assignment and automated identification. Manual assignment led to a set of categories that was more specific to body listening and self-management than those automatically identified through the MetaMap API, but manual classification can be time-consuming. Thus, we now consider ways to improve the classification methods employed in this study.

First, it might be useful to try to reduce the need for additional labor by engaging contributors in the creation of a taxonomy at the time of tag creation and use, which may also improve the specificity of tags and participants’ memories of the content shared [42]. Examples of hybrid taxonomy-folksonomy approaches exist in the literature [43,44]. We might also assist users to tag content by providing recommendations generated through a variety of techniques, including tag cooccurrence, content-based, graph-based, and clustering- or topic-based methods [45]. However, it is important to consider whether the provision of tags might stifle creativity and prevent users from making a greater effort to fully elucidate their thoughts using the most appropriate tag. Finally, extant resources might be employed to automatically categorize tags. In previous literature, resources such as YAGO and WordNet have been used to categorize tags [46], to determine semantic relatedness [47], and to turn a folksonomy into a concept hierarchy [48].

### Limitations

This study has various limitations. First, the duration of this study was 10 weeks, and thus topic coverage was limited to the amount of content that could be covered within that span of time. Given this consideration, the seed topics and the social media recruitment strategy were tailored to focus on a set of conditions with a shared symptomatology. Thus, the health management strategies that are reflected in the data are likely to primarily reflect the interests of this population, and it is necessary to conduct other studies with other target populations to better understand their health management needs.

In addition, the size of the tag corpus was small compared with larger and longer-term social networks such as Flickr and Del.icio.us, and the tag base had not reached a point of stabilization. In the future, it would be useful to employ additional strategies to increase the body of tags, which would most likely also result in revisions to the proposed taxonomy. These techniques might include using the contributed tags as seed terms to identify other relevant terms and categories from conventional knowledge resources or conducting additional Guided Explorations focused on related topics and/or targeting different populations.

Finally, this study informed participants from the outset that all data posted in the ThinkSpace would be public. Not everyone is willing to post in public spaces, and thus, the sample likely reflects this bias. However, we believe that the data contributed serves a valuable function to the public because even if people do not post, many of those who registered and also those who did not likely engaged with the content in their own way and were influenced by it. In addition, previous research has shown that there are different types of users and that a minority percentage of “verbose” taggers can produce results that match and even outperform the semantics from an entire dataset [49].

## Appendix

Multimedia Appendix 1 Inter-rater reliability.

Multimedia Appendix 2 Tags and their assigned categories.
