# Supplementary material for: The User Knows What to Call It: Incorporating Patient Voice Through User-Contributed Tags on a Participatory Platform About Health Management
Source: J Med Internet Res. 2017 Sep 7;19(9):e292. doi: 10.2196/jmir.7673 (PMC5608985; doi:10.2196/jmir.7673)
Supplement: Multimedia Appendix 1 [file jmir_v19i9e292_app1.pdf]

Appendix 1

Inter-Rater Reliability

To evaluate the extent to which two coders would be able to independently classify tags using taxonomy, we calculated inter-rater reliability using three types of agreement. Meta-Category Level Agreement was defined as agreement on at least one meta-category per tag. For example, the two coders agreed that *#gratitude* was a Concept and State, but one coded it as a Concept (CN), and the other, a Positive Action (PA). For Meta-Category Level Agreement, we considered such instances as agreement because the two codes shared the same meta-category. In the case of Category Level Agreement, agreement was determined at the category level. One example was *#kneepillow*, which both coders labeled Supplies/Equipment (SE). Lastly, there were situations in which coders agreed on one category assignment, but not another. Such situations were considered agreement according to the previous two measures, but received a penalty of 0.5 under Partial Agreement. One example was *#letitgo*, which one coder classified as a Concept (CN), and the other coder, a Concept and a Positive Action (CN + A).

From the outset, we suspected that we would achieve higher agreement on the meta-categories than on the categories, for which there existed more ambiguity. On the second round of coding, the two coders achieved the 90% target threshold that was set for Meta-Category Level Agreement, but we report the statistics for the other types because they provide insight concerning the extent to which humans might agree on a classification task using this taxonomy.

Agreement Type	%
Meta-Category Level Agreement	91
Category Level Agreement	81
Partial Agreement	70
